# Effects of 20-Hydroxyecdysone on the Reversible Mitochondrial Transhydrogenase in the Tobacco Hornworm, *Manduca sexta*

**DOI:** 10.1673/031.013.15401

**Published:** 2013-12-17

**Authors:** Kurt P. Vandock, Emily C. Perregaux, Brianna M. Consiglio

**Affiliations:** 1 Bayer CropScience LP, 2 TW Alexander Drive, Research Triangle Park, NC 27709; 2 Department of Biology, Houghton College, Houghton, NY 14744; 3 United States Army Reserve, Army Reserve Medical Command APMC, Forest Park, GA 30297

**Keywords:** ecdysone, ecdysone 20-monooxygenase, energy-linked transhydrogenation, *Manduca sexta* development

## Abstract

The reversible, mitochondrial membrane-associated transhydrogenase from the midgut of *Manduca sexta* (L.) (Lepidoptera: Sphingidae) catalyzes hydride-ion transfer between NADP(H) and NAD(H). The effects of ecdysone and 20-hydroxyecdysone were evaluated and compared to both the NADH-NADP^+^ and NADPH-NAD^+^ transhydrogenations. In the direction of NADPH-formation, the developmentally significant transhydrogenations occur as non-energy- or energy-linked reactions. The energy-linked activity couples with either electron transport-dependent NADH or succinate utilization, or ATP hydrolysis by Mg^++^ -dependent ATPase. Upon the addition of ecdysone alone, all energy-linked reactions in the direction of NADPH formation exhibited a notable increase in activity level over the control reaction. The addition of 20-hydroxyecdysone yielded no significant increase in the activity of any of the transhydrogenations. Synergistic addition of both ecdysone and 20-hydroxyecdysone resulted in no significant effect on transhydrogenase activity. The results of this study make evident a relationship between the presence of ecdysone and 20-hydroxyecdysone on the overall activity of *M. sexta* midgut mitochondrial transhydrogenations. The potential mediation of the energy-linked mitochondrial transhydrogenations involved with NADPH synthesis through the developmental relationship of ecdysone and 20-hydroxyecdysone is considered.

## Introduction

The enzyme transhydrogenase (E.C. 1.6.1.1) has been characterized in humans, mammals, insects, bacteria, and parasites (White et al. 2000; Pestov and Shakhparonov 2009; Yousuf et al. 2010). In parasites (e.g., *Hymenolepis diminuta*), transhydrogenase plays a crucial role in metabolism and serves as an additional site for anaerobic phosphorylation (Park and Fioravanti 2006; Mercer-Haines and Fioravanti 2008; Fioravanti and Vandock 2010). In the tobacco hornworm, *Manduca sexta* (L.) (Lepidoptera: Sphingidae), the mitochondrial transhydrogenase has been characterized as a membrane-associated, phospholipid-dependent, and energy-linked enzyme that contributes to NADPH synthesis and larval development (Vandock et al. 2011). With regard to M *sexta*, the mitochondrial transhydrogenase has been shown to facilitate the reversible reaction noted here (Vandock et al. 2008):


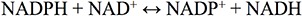


The complete adult life cycle of *M. sexta* consists of five larval stadia lasting approximately 4–6 days each (Kingsolver 2006). At the end of the fifth larval stadium, the insect molts, forms a pupa, and completes its holometabolous metamorphosis. Achievement of post-embryonic development is dependent upon the presence of the molting hormone, ecdysone (Gilbert et al. 2002). This ecdysteroid targets midgut, fatbody, and Malphigian tubules (Smith 1985; Weirich 1997; Gilbert et al. 2002). The P450-dependent ecdysone 20-monooxygenase (E.C. 1.14.99.22; E-20M) catalyzes the conversion of ecdysone (E) to the active form of the molting hormone 20-hydroxyecdysone (20-HE) (Smith et al. 1979; Feyereisen 2005; Lafont et al. 2005). The transitionfrom larval tissues into pupal tissues is characterized by an increase in the level of E-20M activity and a peak in hemolymph ecdysteroid titer (Lafont et al. 2005). During the fifth larval stadium, midgut E-20M activity increases 50-fold between day four and five, corresponding to the increased expression of the shade gene, which encodes E-20M (Mitchell et al. 1999; Gilbert and Rewitz 2009).

Preliminary developmental studies suggest that the role of *M. sexta* transhydrogenase in the energy-linked formation of NADPH may serve as a source of this reducing equivalent which in turn is required for the completion of post-embryonic development (Vandock et al. 2010):


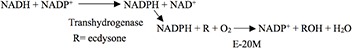


The potential for transhydrogenase to act as a mediator during endocrine-controlled postembryonic development in M *sexta* is evident. In light of this, studies of known flavonoids that act as inhibitors of both E-20M and transhydrogenase have suggested that altering transhydrogenase activity may provide the means for specific control of insect development (Vandock et al. 2012). The following data demonstrates that both the presence of the ecdysteroids E and 20-HE have a direct effect on the energy-linked activities of *M. sexta* mitochondrial transhydrogenations.

## Materials and Methods

### *Manduca sexta* rearing

Fifth instar *M. sexta* were selected and raised on an artificial diet in a standard growth incubator. The environment was controlled under non-diapausing conditions at a photo-period of 16:8 L:D with interruption only for care and feeding. Temperature was controlled at 26° C, and relative humidity maintained at ∼60% (Bell and Joachim 1976).

### Mitochondrial isolation procedure

Mitochondria from 5^th^ instar M *sexta* (Day 4) were extracted by a procedure similar to that described by Vandock et al. (2010). Insects were immobilized on ice prior to isolation of the midguts. Midguts were obtained through careful dissection. Subsequently, the midguts were washed and reserved in a mitochondrial medium (9.0 mL/g tissue) consisting of 250 mM sucrose, 15 mM EDTA, and 10 mM Tris-HCl (pH 7.5). Midguts were homogenized in the mitochondrial medium using a Hielscher Ultrasonic Immersion blender (www.hielscher.com). Cellular debris was separated via differential centrifugation at 480 × *g* for 10 min. The resulting supernatant was centrifuged for 30 min at 9050 × *g* to form the mitochondrial pellet. The pellet yielded from the second spin was resuspended in the mitochondrial medium and washed at 11025 × *g* for 30 minutes, forming the final mitochondrial pellet. This pellet was suspended in TRIS-HC1 buffer (pH 7.5) and stored in a freezer at -20° C until assayed. All steps of the isolation procedure were executed at 4° C.

### Assessments of enzyme activities

Spectrophotometric assessments of transhydrogenase were performed essentially as described by Fioravanti et al. (1992) and Vandock et al. (2008). Activities were measured by following acetylpyridine-NAD(P) (AcPyAD(P)) reduction at 375 nm. For assessment of AcPyAD reduction (NADPH-NAD^+^ reaction), the 1.0 mL assay system contained enzyme, 100 µmol Tris-HCl (pH 7.5), 0.24 µmol NADPH, and 0.60 µmol AcPyAD. Where indicated, 25 µM rotenone was present.

For assessment of AcPyADP reduction, the 1.0 mL assay system contained enzyme, 10 µg bovine serum albumin (BSA), 100 µmol Tris-HCl (pH 7.5), 0.24 µmol NADPH, and 0.60 µmol AcPyAD. Where indicated, the NADH → NADP+ assays contained 25 µM rotenone, 2.0 µM ATP, 3.0 µM MgCL, and 3.0 mM succinate. Rotenone was dispensed in an ethanolic solution, resulting in 1.9% ethanol content in the respective assays. Appropriate ethanol controls were performed.

NADH oxidase and succinate dehydrogenase activities were used as mitochondrial membrane markers. NADH oxidase (E.C. 1.6.99.3) was assessed by measuring NADH disappearance at 340 nm (Fioravanti 1982). The 1.0 mL assay contained enzyme, 10 µg BSA, 100 µmol Tris-HCl (pH 7.5), and 0.24 µmol NADH. . Succinate dehydrogenase (E.C. 1.3.5.1) was assessed essentially by the method of Kmetec and Bueding (1961), as described by Fioravanti (1982), by evaluating potassium ferricyanide reduction at 410 nm. In addition to enzyme and 10 µg BSA, the 1.0 mL assay system contained 100 µmol Tris-HCl (pH 7.5), 0.24 µmol succinate, and 0.60 µmol potassium ferricyanide. All enzyme assessments were performed at 25° C.

### Statistical analyses

Differences between controlled and treated activities were considered significant at *P* ≤ 0.05 using Student's *t-*test. Statistical evaluations were performed using Microsoft Excel 2007 (www.microsoft.com).

### Protein assessment

The protein content of the mitochondrial preparations was determined by the method of Bradford (1976) using BSA as the standard. Inorganic phosphorous content was determined by the method established by Fiske and Subbarow (1925).

### Materials

Organic solvents, BSA, and Tris-HCl were purchased from Fisher Scientific (www.fishersci.com). All other reagents were obtained from Sigma Chemical (www.sigmaaldrich.com). *Manduca sexta* eggs and artificial diet were purchased from Carolina Biological (www.carolina.com). Fifth larval instar specimens were obtained using the methods described by Smith et al. (1979) for the growth and staging of tobacco hornworms. RH-5849 (Rohm and Haas Company; www.dow.com) was a gift from Dr. Martin M. Mitchell, Edinboro University of PA.

## Results

### Transhydrogenase activities

As reported in Vandock et al. (2008), isolated midgut mitochondria from *M. sexta* catalyzed an NADPH-NAD^+^ transhydro-genation as measured by AcPyAD reduction (Table 1). This NADH-forming reaction was the most active transhydrogenation observed. It displayed activity 2-fold over that noted for the greatest NADH-NADP^+^ activity. Inclusion of rotenone led to a slight, but not significant, stimulation of the NADPH-NAD^+^ transhydrogenation (data not shown).

**Table 1.**
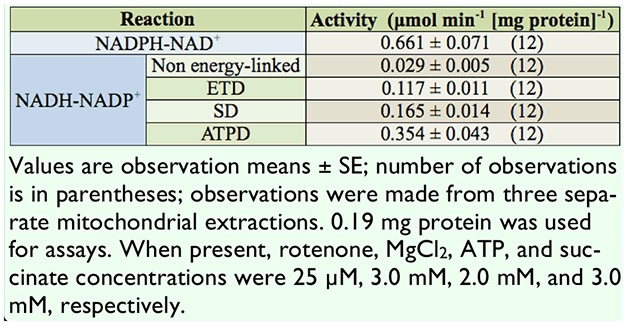
Transhydrogenations catalyzed by isolated *Manduca sexto* midgut mitochondria

The reversibility of the transhydrogenase is evident through observation of an NADH-NADP^+^ transhydrogenation (Table 1). For the NADPH-forming reactions, activity measured in the presence of rotenone was designated as the non energy-linked reaction. In the absence of rotenone, energy-linked NADH-NADP^+^ transhydrogenation resulting from electron transport-dependent NADH oxidation was termed the ETD reaction. Succinate dependent activity in the presence of rotenone was termed the SD reaction. The transhydrogenation driven by ATP hydrolysis via Mg^++^-dependent ATPase was represented by the ATPD reaction. The addition of succinate or ATP plus Mg^++^ significantly increased activity in the presence of rotenone over that noted with rotenone alone in the ETD reaction (Table 1). All energy-linked NADH-NADP^+^ assessments (ETD, SD, and ATPD) were corrected for the activity of the non energy-linked reaction.

Activity levels of the NADPH-NAD^+^ reaction were shown to be more than 4-fold that of the NADH-NADP^+^ reaction. Stimulation through electron transport or ATPase-coupled systems increased this activity, with ATPD displaying the greatest activity level, then SD, followed by ETD (Tablel). No detectable NAD(P)H-dependent reduction of AcPyAD(P) occurred in the absence of enzyme.

### Effects of ecdysone and 20-hydroxyecdysone on the NADPH + NAD^+^—NADP^+^ + NADH transhydrogenation

Activity levels of the NADPH-NAD^+^ transhydrogenation, forming NADH, were measured following the addition of E and 20-HE. The only significant difference from the control reaction was observed with the independent presence of E. Reactions of 20-HE alone and of both ecdysone and 20-hydroxyedysone (E-20-HE) showed no significant increase in the activity level (Table 2). Activity levels of the NADH-NADP^+^ transhydrogenations, forming NADPH, were measured following the addition of E and/or 20-HE (Table 3). Transhydrogenation assessments included the non energy-linked, ETD, SD, and ATPD reactions. The non energy-linked reaction showed the lowest level of activity. Overall, the control ETD reaction showed a 4-fold increase in activity over the non energy-linked reaction. The addition of E resulted in a significant increase compared to the control, 20-HE, and E-20-HE reactions. No significant increase was noted for the control. 20-HE. and E-20-HE reactions.

**Table 2.**
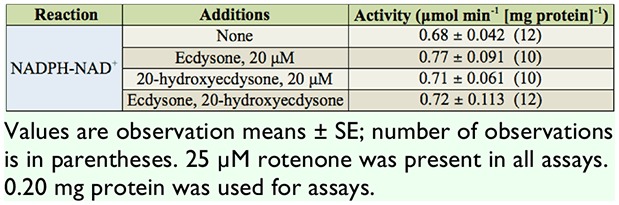
The effects of ecdysone and 20-hydroxyecdysone on the NADPH:NAD^+^ mitochondrial transhydrogenation in *Manduca sexta.*

**Table 3.**
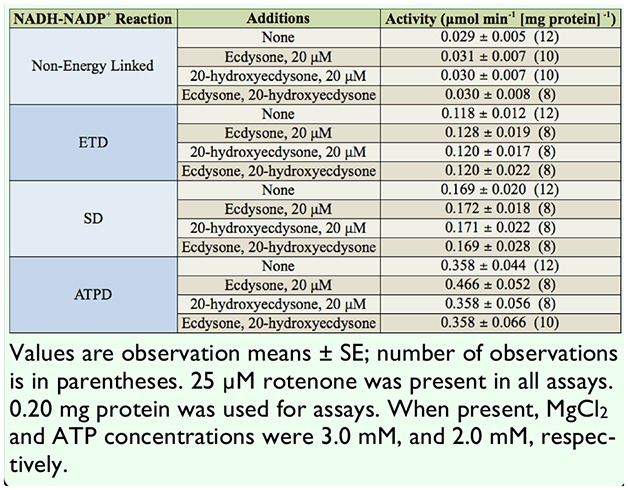
The effects of ecdysone and 20-hydroxyecdysone on the NADH:NADP^+^ mitochondrial transhydrogenation in *Manduca sexta.*

**Table 4.**
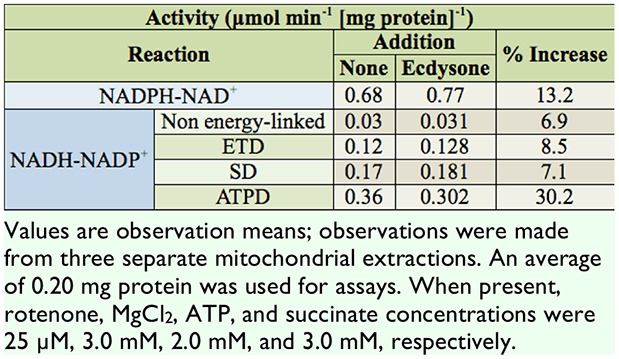
*In vitro* stimulation of the NADPH + NAD^+^ ↔ NADP^+^ + NADH transhydrogenation by ecdysone.

The control SD reaction showed a 5-fold increase in activity over the non energy-linked reaction. Similarly, the greatest increase in activity level was noted for the addition of E No significant increase was noted for the control, 20-HE, and E-20-HE reactions (Table 3).

The control ATPD reaction showed a 12-fold increase in activity over the non energy linked reactions. The addition of E resulted in a marked increase compared to the control, 20-HE, and E-20-HE reactions. No significant increase was noted for the control, 20-HE, and E-20-HE reactions (Table 3).

The effect of E addition on transhydrogenase activity was compared for the non energy-linked, ETD, SD, and ATPD reactions (Table 4). No significant stimulation was observed for the addition of E to the non energy-linked reaction. ETD, SD, and ATPD reactions showed a significant increase when E was added. The greatest stimulation was observed for the ATPD reaction, followed by the SD reaction and then the ETD reaction (Table 4).

## Discussion

The occurrence of a reversible mitochondrial transhydrogenase has been reported in *M. sexta* (Vandock et al. 2008). In M *sexta* midgut and fatbody mitochondria, non-energy-and energy-linked transhydrogenations in the direction of NADPH-formation have been characterized (Vandock et al. 2008, 2010, 2011). The identified energy-linked reactions are driven by ETD, SD, or ATPD. The conversion of E to the active molting hormone, 20-HE, is catalyzed by the NADPH-preferring E-20M, a mitochondrial enzyme predominantly associated with the fifth larval stadium midgut and fatbody tissues of *M. sexta* and other insects (Smith 1985; Weirich 1997). A parallel relationship to NADPH-utilizing E-20M activity was demonstrated by recent biochemical characterization of NADH-NADP^+^ and NADPH-NAD^+^ transhydrogenations in both midgut and fatbody tissues. This parallel relationship was evident in the occurrence of matching peaks of activity for both the transhydrogenase reactions and E-20M activity. These peaks were coincidental with the wandering stage (day 5) of the fifth larval stadium (Mitchell et al. 1999; Vandock et al. 2010).

Potential association of the hormones E and 20-HE with corresponding NADH-NADP^+^ and NADPH-NAD^+^ transhydrogenations appears to be feasible based upon explanations suggested for the protein's mechanism (Vandock et al. 2010). Varying levels of E and 20-HE result from the developmentally significant conversion process executed by NADPH-utilizing E-20M. Ecdysteroid levels fluctuate throughout larval development, with E-20M activity peaking on day 5 (wandering stage). This fluctuation could potentially impact the level of transhydro-genase activity during that same time. As reported in this study, the possibility of a relationship between the two is further supported by the stimulation of mitochondrial transhydrogenase activity from day 4 of the fifth larval instar M *sexta* by the addition of the steroid hormone E. Indeed, in keeping with the NADPH-requiring E-20M, this stimulation was noted for transhydrogenase activity in the direction of NADPH-formation.

Upon the addition of E alone, all energy-linked reactions in the direction of NADPH-formation exhibited a notable increase in activity level over the control reaction. The independent addition of 20-HE yielded no significant increase. Moreover, the NADPH-NAD^+^ energy-linked transhydrogenase activity did not rise when both E and 20-HE were added. The combined addition of E and/or 20-HE had no impact on the activity level of the non energy-linked reactions. Significant stimulation of M *sexta* mitochondrial transhydrogenase is apparent only in the presence of E independently. Similar to the energy-linked NADPH-NAD^+^ transhyrdogenations, the only substantial increase was observed in the reverse direction, NADH-NADP^+^ transhydrogenations, with the addition of E.

The mitochondrial transhydrogenase in *M. sexta* is non-specifically stimulated by the developmental hormone E. It was noted that the addition of E increases the reaction rate of both the NADH-NADP^+^ and NADPH-NAD^+^ transhydrogenations. A preference for NADPH-formation is necessary to facilitate the conversion of E to the active form of the hormone, 20-HE, a process critical for larval insect development (Weirich 1997; Mitchell et al 1999; Gilbert and Rewitz 2009). The effects of E on the mitochondrial transhy-drogenase could be part of a negative feedback mechanism serving as a pathway to supplement the required NADPH. The presence of 20-HE combined with E notably inhibits the stimulatory effect of E. The result is no measurable increase of activity. The fact that this relationship was observed suggests that the activity of the transhydro-genase is indeed linked to endogenous levels of both E and 20-HE. In light of these findings, apparent association of the mitochondrial transhydrogenase to E and 20-HE levels promotes sexual maturity in M *sexta.*

The data from the present study demonstrates that E and 20-HE may be important components related to the mediation of the energy-linked transhydrogenations involved with NADPH synthesis. Energy expenditure required for the energy-linked transhydro-genations (i.e., ETD, SD, ATPD reactions) is substantial. Considered collectively, it would appear that the larval development of M *sexta* relies upon the capability of E to stimulate transhydrogenase, among other related mitochondrial enzymes (NADH oxidase, succinate dehydrogenase, Mg^++^-dependent ATPase). This proposed stimulation would provide sufficient energy for the conversion of NADPH, thereby allowing oxidation of E to 20-HE. The presence of 20-HE inhibits the stimulatory effects of E on the NADPH-NAD^+^ reactions. Subsequently, the relative formation of NADPH appears to be impacted by the amount of each respective ecdysteroid in response to the specific developmental stage of the organism.

Data related to the energy-linked transhy-drogenations provides evidence for the possible dependence of transhydrogenase on a proton motive force present from the mitochondrial electrochemical gradient. This would imply that once a critical threshold for the electrochemical gradient is reached, the transhydrogenase would operate in the formation of NADPH. Reaching this critical threshold would require significant energy expenditure because protons must be actively transported against the concentration gradient via Mg^++^ -dependent ATPase. Energy-linked transhydrogenations, such as the ATPD reaction, would seem to allow the highest level of NADPH-formation due to rapid accumulation of protons. Through this relationship, it is possible that the non energy-linked transhydrogenations exhibit a low level of activity in the direction of NADPH as a result of inadequate proton motive force. Given the steep energy requirements of the energy-linked reactions, it would seem reasonable to conclude that the amount of energy used to produce the required NADPH necessary for 20-HE conversion and successful adult development is carefully regulated. Considering the effect of E and 20-HE on transhydrogenase activities, this study supports such regulation via the involvement of energy-linked transhydrogenations and the conversion of E to 20-HE.

The parallel relationship demonstrated by the NADPH-utilizing E-20 M activity and NADH-NADP^+^ and NADPH-NAD^+^ transhydrogenations suggests that the ecdysteroids E and 20-HE have a direct impact on these mitochondrial transhydrogenase activities. For the NADPH-NAD^+^ energy-linked reactions, only the singular addition of E resulted in significant stimulation of *M. sexta* mitochondrial transhydrogenase. Consequently, the simultaneous addition of both 20-HE and E, as well as the addition of 20-HE alone, yielded no significant increase in the mitochondrial transhydrogenations. Increased activity levels in both the NADPH- NAD^+^ and NADH-NADP^+^ transhydrogenations supports non-specific stimulation of transhydrogenase by E and was demonstrable in this study. Non-energy-linked reactions displayed no notable increase in activity. The fact that 20-HE is capable of inhibiting the stimulatory effects of E reveals that only E is capable of impacting the activity level of the *M. sexta* transhydrogenase. The results suggest that transhydrogenase activity and endogenous levels of E and 20-HE are closely associated with one another and act synergistically to mediate energy-linked NADPH-NAD^+^ transhydrogenations. The substantial energy expenditure required for adult development is provided via transhydrogenase activity in the formation of NADPH. This activity may be controlled in accordance with the mitochondrial electrochemical gradient, which is successfully attained by the energy-linked reactions.

The results of this finding make evident a relationship between the presence of E and 20-HE on the overall activity of *M. sexta* midgut mitochondrial transhydrogenations. While the exact physiological impacts of this relationship throughout the life cycle of *M. sexta* require further investigation, this study supports the notion that inner mitochondrial membrane-associated transhydrogenase may indeed be involved with endocrine control of post-embryonic development in *M. sexta.*

## Abbreviations:

20-HE,: 20-hydroxyecdysone;
AcPyAD(P),: acetylpyridineNAD(P);
RT-PCR,: Mg^++^-dependent ATP hydrolysis;
E,: ecdysone;
E-20M,: ecdysone 20-monooxygenase;
ETD,: electron transport-dependent NADH oxidation;
SD,: succinate dependent oxidation
